# Optimized invisibility cloaks from the Logarithm conformal mapping

**DOI:** 10.1038/srep38443

**Published:** 2016-12-06

**Authors:** Chunhui Zhu, Lijun Liu, Zhengyong Song, Qing Huo Liu

**Affiliations:** 1 The Department of Electronic Science, Xiamen University, Xiamen, 361005, China; 2 The Department of Automation, Xiamen University, Xiamen, 361005, China; 3 The Department of Electrical and Computer Engineering, Duke University, Durham, NC 27708, USA

## Abstract

Invisibility cloaks designed from the coordinate transformation method have attracted increasing interest recently. Conformal transformation optics scheme leads to cloaks that possess isotopic media, thus provides a prospective way to facilitate easier realization. Reducing the maximum value of the refractive index required by the cloaks is very important in practical imple- mentation. This letter studies on how the parameters in the logarithm conformal mapping control the cloaking effect. The optimized invisibility cloaks are designed. The maximum values of the refractive index required from the first kind and the second kind of logarithm conformal mappings are reduced to 9.779 and 12.936, respectively.

Two pioneer works about invisibility cloaks designed using transformation optics (TO) theorem are reported in 2006^1^,^2^. Since then, devices designed based on TO have attracted significant attention^3^,^4^,^5^,^6^,^7^,^8^,^9^,^10^,^11^,^12^,^13^,^14^. The fundamental of TO is the invariant of Maxwell’s equations under coordinate transformation^15^,^16^. Among all the related techniques, the methods based on conformal transformation optics (CTO)^17^ show their great advantages since the media designed are isotopic. This makes the experimental realizations more feasible. Therefore, CTO has been used in different kinds of designs including wave bend device^9^, directional emitter^18^, unidirectional radiations^12^, plasmonic nanostructures^19^,^20^,^21^ and invisibility cloak^22^,^23^. CTO can also offer a new approach to investigate a physical system’s properties. For example, Huidobro *et al*.^24^ presented an analytical model to study the optical response of subwavelength graphene gratings; Aubry *et al*.^24^ investigated the interaction between plasmonic nanowire dimers; Huidobro *et al*.^25^ and Li *et al*.^26^ proposed efficient strategies to control the flow of surface plasmon polaritons; Pendry and Luo *et al*.^27^,^28^ proposed an analytical solution for interactions at the nanometer scale, which can be used to analyzed accurately the nonlocality in 3D plasmonic systems; Kraft *et al*.^29^ calculated analytically the electromagnetic properties of an spheroid.

In order to ease the experimental fabrication of invisibility cloaks, it is very important to reduce the maximum value of the refractive index required. The Zhukowski conformal mapping (ZCM) is used by Leonhardt^1^ to design an invisibility cloak combined with the Kepler potential, where the maximum refractive index required is 36. By combining the ZCM with two kissing mirrored Maxwell’s fish eye lenses, the maximum refractive index required is reduced to 13^30^. Xu and Chen^22^ design the invisibility cloaks by two logarithm conformal mappings (LCM) combined with two kissing mirrored Maxwell’s fish eye lenses, where the maximum refractive index of the materials are 9.838 and 22.043, respectively.

In this work, we study on how the parameters in the LCM control the cloaking effect. When designing an invisibility cloak from the ZCM given by *w* = *z* + *a*^2^/*z*, the area of the cloak is controlled by only one parameter *a*^1^,^30^. Though there are two parameters to be decided in the cloak from the LCM, we note that there is a similar property. Accordingly, the size of the invisible area could be controlled flexibility by changing only one parameter. The optimized invisibility cloaks are designed. We show that the maximum value of the refractive index of an invisivility cloak from the second kind of LCM can be reduced to 12.936. This is just a little bigger than half of 22.043 which is given by Xu and Chen^22^. From the first kind of LCM, we also reduce the maximum value of the refractive index to 9.779.

## Results

Before addressing our results, we briefly recall the cloaking effect brings by the LCM. As a generalization from the work by Xu and Chen^22^, we give the definition of the LCM as


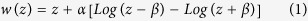


where *α* is a positive real number. When *β* < *α*/2 and *β(β* − 2*α*) > 0, we refer to equation (1) as the first kind and the second kind of LCM, respectively. *z* plane is the physical space and *w* plane is the virtual space. By equation (1), the physical space is mapped into two Riemann sheets in the virtual space.

The mapping from equation (1) is introduced here in short. Details can be found in Xu and Chen’s work^22^ and the supplementary information therein. The two Riemann sheets in *w* plane are connected by a branch cut, which is the finite straight line connecting *w(z*_1_) and *w(z*_2_). 
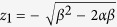
 and *z*_2_ = −*z*_1_ are subject to *w*′(*z*_1_) = 0 and *w*′(*z*_2_) = 0. According to equation (1), the branch cut in *w* plane is mapped from a closed curve in *z* plane. The outside of this closed curve in *z* plane is mapped into the first Riemann sheet in *w* plane. Denote *w* = *u* + *iv*. Then this first Riemann sheet is infinite both in *u* and *v* directions. The inside of the closed curve in *z* plane is mapped into the second Riemann sheet, which is infinite in *u* direction and finite in *v* direction. And the width is 2*απ* in *v* direction.

Followed the design by Xu and Chen^22^, in the virtual space, two kissing mirrored Maxwell’s fish eye lenses with PEC boundary are put in the second Riemann sheet. When the light rays at an eigen-frequency *f*_*l*_ enter from the first Riemann sheet into the second Riemann sheet, this design will guide them to return to the first Riemann sheet conserving the positions and directions. Denote *r* = |*w*_1_ − *w*_2_|/2. Then the radius of each Maxwell’s fish eye lens is *r*. For the first kind of LCM, the centers of the two lenses locate at (0, *r*) and (0, −*r*), respectively, when *r* ≤ *απ*/2. For the second kind of LCM, the centers of the two lenses locate at (*r*, 0) and (−*r*, 0), respectively, when *r* ≤ *απ*. Here the eigen-frequency is 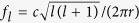, where *c* is the speed of light in vacuum and *l* is an integer. Correspondingly, in the physical space, all the light rays impinging at *f*_*l*_ will propagate around the PEC boundary in *z* plane. This forms an area that the light rays cannot reach, which is the invisibility area brings by the LCM.

**Figure 1:**
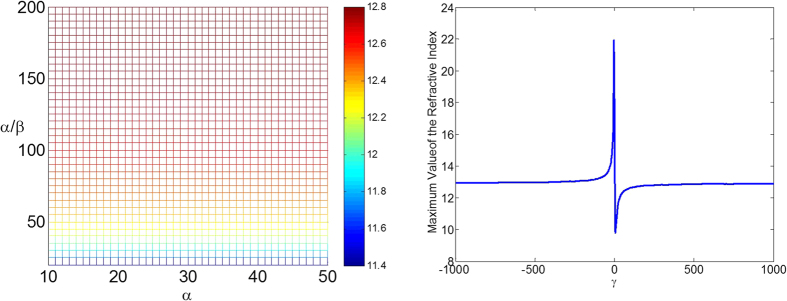
The maximum values of the refractive index of the invisibility cloaks. The left one shows the cases for different *α* and *β*; the right one shows the cases for different *γ*.

We show the maximum values of the refractive index required when *α* and *β* vary in the left of Fig. 1. It is noted that when the ratio between *α* and *β* is invariant, the range of the refractive index profile is also invariant. We will give the explanation of this for the second kind of LCM below. The case for the first kind of LCM can be explained in the same manner. The refractive index distribution from the second kind of LCM descripted above can be expressed as ref. 22


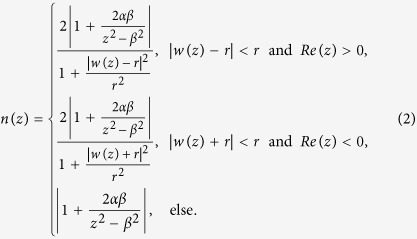


Let us rewrite equation (1) as *w(z*) = *z* + *α[Log(z/β* *−* 1) − *Log(z/β* *+* 1)] and denote 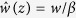 and 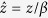. Then equation (1) becomes


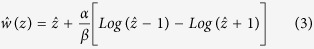


Denote *γ* = *α/β*, then there is


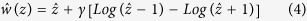


Consider the forms of *w*_1_, *w*_2_, the refractive index profile *n(z*) and *β* < 0, equation (2) can be rewritten as


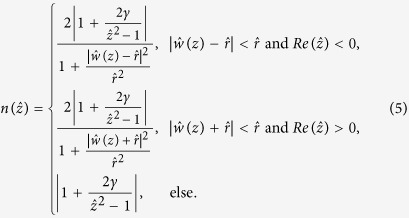


We call the 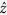 plane the reference physical space, and call the 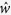 plane the reference virtual space below.

It can be seen from equation (5) that the refractive index distribution in the reference physical space 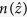 is only decided by *γ. n(z*) in the physical space equals 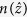 based on 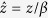 in the reference physical space. Therefore, when *β* varies and *γ* is invariant, the cloak area in the physical space is proportional to *β*, but the range of the refractive index profile does not change. So we can first design the cloak according to requirement by only considering one parameter *γ* based on the conformal transformation in equation (4), which is from the reference physical space to the reference virtual space. And then the real cloak area can be obtained by adjusting *β* according to requirement correspondingly. The right of Fig. 1 shows how the maximum value of the refractive index changes when *γ* varies between −1000 and 1000.

**Figure 2:**
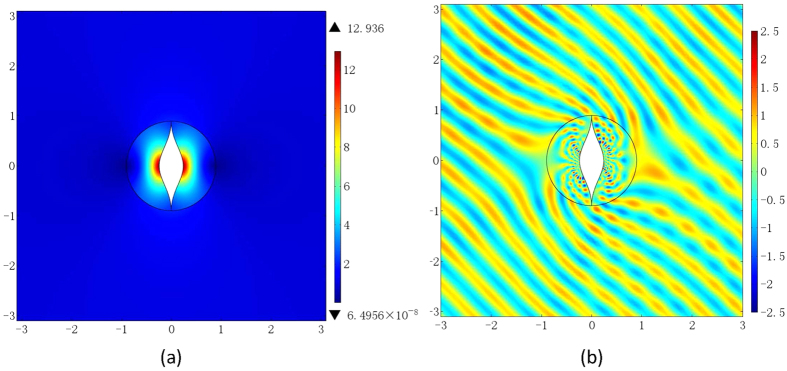
The optimized refractive index distribution and the electric field pattern from the second kind of LCM. (**a**) The refractive index distribution when *α* = 40 and *β* = −0.01. (**b**) The electric field pattern, when a plane wave incidents from 45° angle at frequency *f*_*l*_ with *l* = 20.

*α* = 4 and *β* = −1 are adopted by Xu and Chen^22^ and the range of refractive index profile obtained is from 0 to 22.043. It is observed that this is the case from the right of Fig. 1 when *γ* = −4. This can be improved by changing *γ*. Figure 2(a) shows the refractive index distribution when *α* = 40 and *β* = −0.01. The refractive index profile ranges from 0 to 12.936 in this case, where the maximum value required is only a little bigger than half of 22.043. Figure 2(b) displays the electric field pattern for the invisibility cloak from the proposed conformal mapping when a plane wave incidents from 45° angle at the eigen-frequency *f*_*l*_ when *l* = 20.

**Figure 3:**
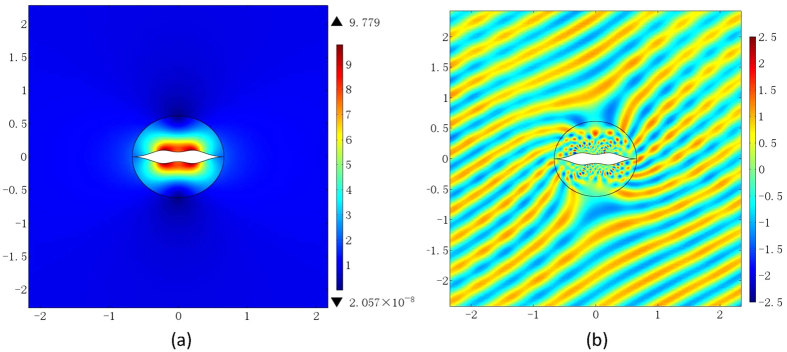
The optimized refractive index distribution and the electric field pattern from the first kind of LCM. (**a**) The refractive index distribution when *α* = 1 and *β* = 0.21. (**b**) The electric field pattern, when a plane wave incidents from 120° angle at frequency *f*_*l*_ with *l* = 18.

*α* = 4 and *β* = 1 are also adopted by Xu and Chen^22^ and the range of the refractive index profile obtained is from 0 to 9.839, where *γ* = 4. We find that this can also be slightly improved by adopting *γ* = 4.71, the maximum value of the refractive index in this case in 9.779 as shown in Fig. 3(a). Figure 3(b) displays the electric field pattern for the invisibility cloak from the proposed conformal mapping when a plane wave incidents from 120° angle at the eigen-frequency *f*_*l*_ when *l* = 18.

It should be noted from equation (5) that the minimum values of the refractive index before and after the optimization are both 0 when 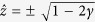, which are two points locating at the inner boundary of the cloak. Therefore, the proposed method could not help to get rid of the difficulty regarding the near-zero refractive index.

## Discussion

In conclusion, we have studied on how to control the invisibility cloak with the parameters from the LCM. By introducing the reference physical space and the reference virtual space, we explain that the maximum value of the refractive index required is only decided by the ratio of *α/β*, and the area of the invisibility area is proportional to *β*. Based on this observation, the invisibility cloaks from the LCM are optimized. The maximum value of the refractive index profile for the first kind of LCM is reduced to 9.779 and that for the second kind of LCM is reduced to 12.936.

**Figure 4:**
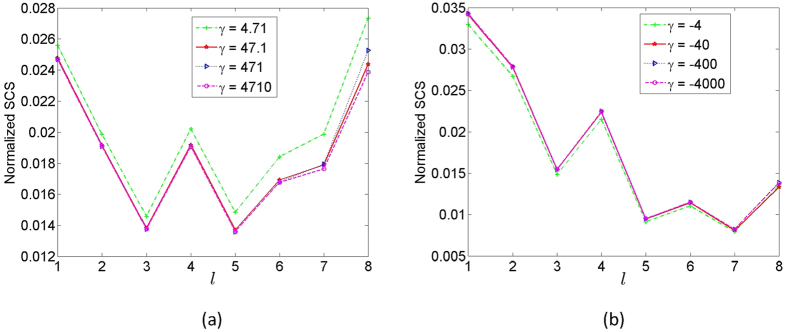
The normalized SCS. (**a**) The first kind of LCM. (**b**) The second kind of LCM.

As discussed by Yaroslav *et al*.^16^, the scattering cross section (SCS) from a conformal transformation is mainly decided by the cutoff in the cloak size. Figure 4(a) and (b) show the SCS normalized by the size of the cloaks. For the first kind of LCM, the cloaks are all cut off at 77|*z*_1_|. For the second kind of LCM, the cloaks are all cut off at 63|*z*_1_|. As expected, it can been detected that the normalized SCS is not affected very much by the parameter *γ*.

## Additional Information

**How to cite this article**: Zhu, C. *et al*. Optimized invisibility cloaks from the Logarithm conformal mapping. *Sci. Rep.* **6**, 38443; doi: 10.1038/srep38443 (2016).

**Publisher's note:** Springer Nature remains neutral with regard to jurisdictional claims in published maps and institutional affiliations.
